# Simulated ischaemia/reperfusion impairs trophoblast function through divergent oxidative stress- and MMP-9-dependent mechanisms

**DOI:** 10.1042/BSR20240763

**Published:** 2024-11-21

**Authors:** Aaron Barron, Jetro J. Tuulari, Linnea Karlsson, Hasse Karlsson, Gerard W. O'Keeffe, Cathal M. McCarthy

**Affiliations:** 1 Department of Anatomy and Neuroscience, University College Cork, Cork, Ireland; 2 Department of Pharmacology and Therapeutics, University College Cork, Cork, Ireland; 3 FinnBrain Birth Cohort Study, Turku Brain and Mind Centre, Department of Clinical Medicine, University of Turku, Turku, Finland; 4 Department of Psychiatry and Turku Brain and Mind Centre, University of Turku and Turku University Hospital, Turku, Finland; 5 Turku Collegium for Science, Medicine and Technology, University of Turku, Turku, Finland; 6 Centre for Population Health Research, University of Turku, Turku University Hospital, Turku, Finland; 7 Department of Clinical Medicine, Paediatrics and Adolescent Medicine, Turku University Hospital and University of Turku, Turku, Finland; 8 Cork Neuroscience Centre, University College Cork, Cork, Ireland

**Keywords:** cell invasion, cell migration, ischemia, metalloproteases, oxidative stress, preeclampsia

## Abstract

Early-onset pre-eclampsia is believed to arise from defective placentation in the first trimester, leading to placental ischaemia/reperfusion (I/R) and oxidative stress. However, our current understanding of the effects of I/R and oxidative stress on trophoblast function is ambiguous in part due to studies exposing trophoblasts to hypoxia instead of I/R, and which report conflicting results. Here, we present a model of simulated ischaemia/reperfusion (SI/R) to recapitulate the pathophysiological events of early-onset pre-eclampsia (PE), by exposing first trimester cytotrophoblast HTR-8/SVneo cells to a simulated ischaemia buffer followed by reperfusion. We examined different ischaemia and reperfusion times and observed that 1 h ischaemia and 24 h reperfusion induced an increase in reactive oxygen species (ROS) production (*P*<0.0001) and oxygen consumption rate (*P*<0.01). SI/R-exposed trophoblast cells exhibited deficits in migration, proliferation, and invasion (*P*<0.01). While the deficits in migration and proliferation were rescued by antioxidants, suggesting an ROS-dependent mechanism, the loss of invasion was not affected by antioxidants, which suggests a divergent ROS-independent pathway. In line with this, we observed a decrease in MMP-9, the key regulatory enzyme necessary for trophoblast invasion (*P*<0.01), which was similarly unaffected by antioxidants, and pharmacological inhibition of MMP-9 replicated the phenotype of deficient invasion (*P*<0.01). Collectively, these data demonstrate that I/R impairs trophoblast migration and proliferation via a ROS-dependent mechanism, and invasion via an ROS-independent loss of MMP-9, disambiguating the role of oxidative stress and providing insights into the response of trophoblasts to I/R in the context of early-onset PE.

## Introduction

Pre-eclampsia (PE) is a common hypertensive disorder which complicates 3–5% of pregnancies, and is a leading cause of maternal mortality and morbidity [1–5]. PE equally has deleterious consequences for the fetus—it is a leading cause of neonatal death and a major risk factor for comorbid obstetric complications, and long-term cardiovascular and neurodevelopmental morbidity in exposed offspring [6–9]. Early-onset PE is thought to develop due to a failure of extravillous cytotrophoblasts (EVTs) of the developing placenta to appropriately invade the uterus in the first trimester [3,10]. During pregnancy, these cells undergo an epithelial-to-mesenchymal-like transition (EMT) and become highly invasive cells that penetrate myometrium and spiral arteries, promoting their remodelling to facilitate the increased blood flow demanded by the placenta in the second and third trimesters [10,11]. In early-onset PE, however, there is evidence of defective placentation mediated in part by inadequate trophoblast invasion, and thus insufficient spiral artery remodelling [12,13]. This results in fluctuations in blood flow to the placenta, which may lead to placental ischemia/reperfusion (I/R) injury and consequent oxidative stress. These disruptions in placental blood flow are thought to be responsible for the increased placental secretion of soluble factors into the maternal circulation that drive the pathophysiology of the disease [3,14]. Thus the role of I/R in the placenta has been of immense interest to researchers examining the mechanisms of pathogenesis in early-onset PE.

Most studies have modelled PE *in vitro* by using either primary human EVTs, or more commonly the first trimester cytotrophoblast cell line HTR-8/SVneo, to study the functional properties of human trophoblast cells—proliferation, migration, and invasion [15]. These studies most frequently involve exposure to hypoxia, either directly in the form of reduced O_2_ concentration, or treatment with CoCl_2_ to stabilize HIF-1α and HIF-2α, and have yielded highly discordant findings. Many studies report that hypoxia increases trophoblast migration and invasion [16–18], while others have reported the opposite [19], and this discordance cannot be solved by stratifying the studies by cell type, hypoxic model, or timing of exposure. As well as differences in their findings, there are three fundamental problems with these models in the context of PE. Firstly, while placental hypoxia is often assumed in PE, there is no evidence for this. Although abnormal uteroplacental perfusion is a well-documented element of PE [12,13], malperfusion is not equivalent to hypoxia, which must be defined by oxygen concentration dropping below the minimum metabolic requirements of the cells, and placental O_2_ has been found to be unchanged or even elevated in PE (hyperoxia) [20]. In fact, exposing cells to 2–5% O_2_ is more reflective of the physiological environment of the first trimester in a normal pregnancy than it is in PE [21]. While these studies have led to valuable insights into the effects of low O_2_ on trophoblast function, they cannot be interpreted as a model of PE.

The second concern is that most studies fail to incorporate reoxygenation/reperfusion in their model. Reperfusion is not the end of the physiological insult, but rather an essential part of it [22]. It has been shown that hypoxia, hypoxia/reoxygenation, and CoCl_2_ each results in a fundamentally different metabolomic profile in trophoblasts, suggesting these models cannot be seen as equivalent [23]. Furthermore, increased invasion reported in many hypoxia studies is incompatible with our current understanding of shallow trophoblast invasion in PE. In fact, only when hypoxia chamber studies are dichotomised into those that examine hypoxia alone or those that include a reoxygenation period afterwards, does a pattern finally emerge in the literature whereby hypoxia most commonly increases trophoblast migration and invasion [24–29], while hypoxia/reoxygenation decreases migration and invasion [30–35]. Lastly, even when studies include a reoxygenation period, they often fail to mimic the fluctuating restriction of nutrients, metabolic substrates and ions that also occurs during I/R, by focusing solely on oxygen. Collectively, the failure to see hypoxia and I/R as distinct phenomena has ambiguated our understanding of the molecular pathophysiology of both conditions, and it is only the latter that is believed to occur in PE.

Due to these disparities, the exact role of I/R and the contribution of consequent oxidative stress, remains elusive. Placental oxidative stress is a well-documented component of PE, where there is extensive evidence for increased reactive oxygen species (ROS), oxidative cell damage, and mitochondrial dysfunction [36]. This has been postulated to have a causal role in the pathogenesis of PE, yet it is difficult to demonstrate experimentally and has been called into question by the failure of the antioxidant vitamin C and E trials to prevent the occurrence of PE [37]. Thus, the exact contribution of oxidative stress to trophoblast dysfunction in PE, and to the disorder more broadly, is still unclear, and this is a major gap in our understanding of the progression of the disease.

One solution may be an approach common to cardiomyocyte research: simulated ischemia/reperfusion (SI/R), the use of a buffer which deprives cells of metabolic substrates while incorporating compounds that induce acidosis, lactate production, hyperkalaemia, mitochondrial starvation, and impaired mitophagy, followed by reperfusion to induce oxidative stress [38–41]. Despite recapitulating a great number of the cellular components of I/R injury seen in PE and being highly reproducible, flexible and inexpensive, to date few studies have applied this approach within PE research. To the best of our knowledge, only one study has used this model in HTR-8/SVneo trophoblasts and demonstrated that I/R induces apoptosis, but the authors did not examine trophoblast migration, proliferation, or invasion. Importantly, the authors showed that these apoptotic effects, and the molecular mechanisms involved, were equivalent when using either the SI/R model or the conventional hypoxia/reoxygenation model [42].

Overall, the effect of I/R on trophoblast function, and to what extent ROS are involved, remains elusive. To address these questions, in this current study we firstly sought to validate the use of an SI/R model in HTR-8/SVneo trophoblasts, with optimized ischaemia and reperfusion times, which tend to vary significantly across studies in other contexts. We next studied the effects of this SI/R model on oxygen consumption rate, migration, proliferation, and invasion, and used antioxidant treatments to discern the potential role of ROS in any observed functional deficits.

## Materials and methods

### Cell culture

Human first trimester cytotrophoblast HTR-8/SVneo cells (ATCC) were cultured in Roswell Park Memorial Institute (RPMI) 1640 medium (Gibco), supplemented with 2 mM L-glutamine, 1% penicillin-streptomycin, 1 mM sodium pyruvate, and 10% FBS (all from Sigma–Aldrich). 2 × 10^6^ cells were added to a T75 culture flask (Stardest) and maintained in a humified atmosphere at 37°C and 5% CO_2_. Media was changed every 3 days and cells were passaged and/or seeded for experiments once they reached ∼ 80% confluence. All cells used were at passages P9–P16.

### Simulated ischemia/reperfusion and pharmacological treatments

**Figure 1:**
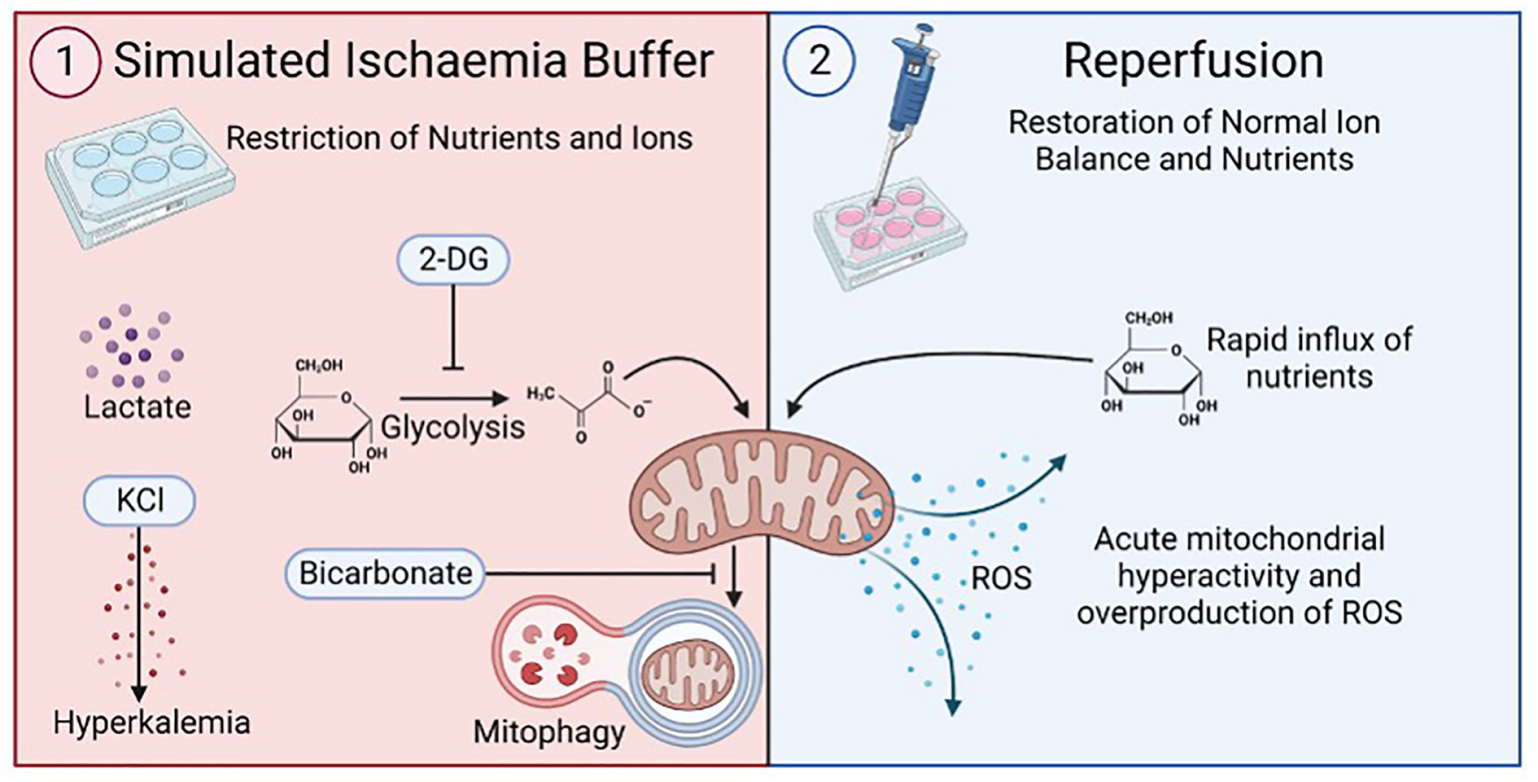
Simulated ischaemia/reperfusion Schematic illustration of simulated I/R injury used in the current study. Cells are incubated in a simulated ischaemia buffer, comprising a physiological salt solution lacking essential nutrients and supplemented with sodium-l-lactate to mimic lactate accumulation, potassium chloride to induce hyperkalaemia, 2-deoxyglucose to inhibit glycolysis and starve mitochondria, and sodium bicarbonate to inhibit mitophagy. Upon reperfusion, normal ion balance is restored, and a rapid influx of metabolic substrates induces mitochondrial hyperactivity and excessive production of ROS. Figure made using BioRender.com. 2-DG = 2-deoxyglucose, KCl = potassium chloride, ROS = reactive oxygen species.

To model I/R, cells were plated and allowed to be attached for 24 h in the growth media described above. Seeding densities depended on the experiment and are specified below. Following this, media was removed, and cells were washed once in HBSS (Sigma). Simulated ischaemia buffer was added to each well and cells were incubated for 1 h at 37°C and 5% CO_2_ [38,40–42]. Simulated ischaemia buffer was prepared and filter-sterilised on the day of exposure as per Table 1. After 1 h incubation, buffer was removed entirely and replaced with pre-warmed complete media (Figure 1). Control wells were also given a media change at this point. Where indicated, some experimental groups received a single treatment of 500 μM N-acetyl cysteine (NAC) (Sigma–Aldrich), 1 mM L-ergothioneine (ERG) (Tetrahedron) or 10 nM MMP-9 inhibitor CAS 1177749-58-4 (Santa Cruz Biotechnology, ID_50_ = 5 nM). In all experiments, control wells were treated with an equal volume of the vehicle (dH_2_O for antioxidants NAC and ERG, DMSO for MMP-9 inhibitor).

**Table 1:** Composition of simulated ischaemia buffer

Component^*^	Molecular Formula	Concentration (mM)
Sodium Chloride	NaCl	118
Sodium Bicarbonate	NaHCO_3_	24
Monosodium Phosphate	NaH_2_PO_4_	1
Calcium Chloride	CaCl_2_	2.5
Magnesium Chloride	MgCl_2_	1.2
Potassium Chloride	KCl	16
Sodium-L-Lactate	NaC_3_H_5_O_3_	20
2-Deoxyglucose	C_6_H_12_O_5_	10

* The buffer prepared in dH_2_O and filter-sterilised on day of use.

### ROS measurement

Cells were plated at a density of 12,500 cells/cm^2^ in a 24-well plate. At the experimental end points indicated in the figure legends, ROS levels were assessed with the fluorescent live-cell dye CellROX™ Green Reagent (Invitrogen), which is oxidised by intracellular ROS to a product which fluoresces green in proportion to cellular ROS concentration. As per manufacturer’s guidelines, 5 μM CellROX™ Green Reagent was added to the media in each well and the plate was then incubated at 37°C for 30 min, washed once with 10 mM PBS and imaged live in PBS at 20× magnification under FITC fluorescent channel, with an Olympus IX71 inverted microscope. Five non-overlapping fields were imaged per well, using a DP72 camera. The mean fluorescence intensity of five cells per field minus adjacent background was measured using ImageJ.

### Cytotoxicity

Cytotoxicity was measured using the CyQUANT™ LDH Cytotoxicity Assay Kit (Invitrogen), which is a colorimetric assay based on extracellular lactate dehydrogenase (LDH) activity as a proxy for cell membrane damage. As per manufacturer’s guidelines, cell culture media was collected at each experimental endpoint, centrifuged to remove any suspended cells and debris, and the supernatant was used for the LDH assay. A 50 μl medium was combined with 50 μl reaction mixture in a flat-bottomed 96-well plate and incubated for 30 min in darkness at room temperature. A 50 μl stop solution was used to stop the reaction, and absorbance was measured at 680 nm and subtracted from absorbance at 490 nm.

### Oxygen consumption rate

Oxygen consumption rate (OCR) was measured with the Resipher system (Lucid Scientific). Cells were plated at 20,000 cells per well in 32 wells of a 96-well plate (wells A3:H4 and A9:H10, as per the positioning of sensing probes). After 2 h, the lid was replaced with a Resipher sensing lid and connected to the Resipher at 37°C and 5% CO_2_ for continual OCR recordings. The 24 h of baseline recordings were acquired prior SI/R. Cells were washed with HBSS and exposed to SI/R for 1 h, at which point the ischaemia buffer was removed and complete media was added to each well. Cells were reconnected to the Resipher at 37°C and 5% CO_2_ and a further 24 h of OCR recordings were collected. At experimental end-point, cells were lysed in 1X RIPA buffer and total protein quantified by bicinchoninic acid (BCA) assay, and OCR values normalized to protein content (μg/ml) per well. OCR data were collected in Lucid Lab® analysis suite and exported for analysis. All experimental OCR values were normalised to the last pre-intervention baseline value per well, to account for well-to-well baseline variability.

### Migration

Trophoblast cell migration was measured using the scratch wound assay [43]. HTR-8/SVneo cells were plated at a density of 75,000 cells/cm^2^ in a 12-well plate and grown until confluent for 48 h. Following SI/R (if applicable), a single, straight wound was created through the cell monolayer with a P200 pipette tip, and media was changed. Cells were then treated with antioxidant or MMP-9 inhibitor. The wound was imaged using phase contrast microscopy at 10× magnification with an Olympus IX71 inverted microscope at three distinct locations along the scratch in each well at 0 and 24 h post-scratch. The difference in wound width between 0 and 24 h timepoints was measured using ImageJ, which represented cell migration.

### Colony formation assay

Trophoblast cell proliferation was measured by colony formation assay [44,45]. HTR-8/SVneo cells were seeded at low density (125 cells/cm^2^) in a 12-well plate, and after 24 h were exposed to SI/R and antioxidants or MMP-9 inhibitor treatments. Cells were incubated at 37°C and 5% CO_2_ for 10 days (5 cell cycles), where full media and treatments were replaced every 3 days. At experimental end-point, media was aspirated, and cells were washed once in PBS, fixed in 4% paraformaldehyde (PFA), and stained with 0.5% cresyl violet at room temperature for 15 min. Cresyl violet was decanted, and wells were washed in dH_2_O, at which point visible cell colonies were evident. Plate was imaged and the number of colonies per well counted with ImageJ.

### Laminin transwell invasion assay

Trophoblast cell invasion was measured by laminin transwell invasion assay [43]. HTR-8/SVneo cells were grown until confluent for 48 h. Following exposure to SI/R (if applicable), cells were enzymatically detached with trypsin-EDTA for 5 min at 37°C, which was then neutralized with equal volume of complete media and centrifuged at 200 × g for 5 min, and the supernatant discarded. Cells were resuspended in serum-free media, counted and used to prepare a cell suspension with a concentration of 5 × 10^5^ cells/ml. From this, 200 μl (10^5^ cells) was added to the upper chamber of a 8 μm pore size transwell insert which had been pre-coated with 20 μg/ml laminin, and 500 μl complete media was added to the lower chamber. Cells were treated with antioxidants or MMP-9 inhibitor and incubated for 24 h at 37°C and 5% CO_2_. Serum acts as a chemoattractant, promoting cell migration from the serum-free upper chamber, through the laminin-coated transwell, to the lower chamber which contains 10% FBS. After 24 h, transwells were removed, washed twice in 10 mM PBS, and the cells that remained in the upper chamber were removed with a cotton swab. Cells that had invaded the lower surface of the transwell were fixed in 4% PFA, washed twice in PBS, stained for 5 min with DAPI (1:4000), and washed twice more in 10 mM PBS. Stained transwells were imaged at 20× magnification with an Olympus IX71 inverted microscope. Five non-overlapping fields per transwell were captured using a DP72 camera. The number of cells per field of view was quantified in ImageJ.

### ELISA quantification of matrix metalloproteinases

The concentration of matrix metalloproteinases (MMPs) 1, 3, and 9, was measured in the HTR-8/SVneo-conditioned media samples collected at the end of scratch assay experiments, using the MMP 3-Plex Ultrasensitive Kit immunoassay (Mesoscales Diagnostics). The assay was carried out according to manufacturer’s instructions and analysed on the Meso QuickPlex SQ 120. MMP concentrations (pg/ml) were calculated on the Mesoscale Discovery Workbench 4.0 assay analysis software.

### MMP-9 activity

Extracellular activity of MMP-9 was assessed by gelatin zymography, adapted from a protocol by [46]. Conditioned media from scratch assay experiments were mixed with non-reducing sample buffer and loaded on a 10% polyacrylamide gel containing 0.1% (w/v) gelatin, and proteins were separated by polyacrylamide gel electrophoresis (PAGE). Proteins were then renatured in washing buffer [Triton X 2.5% (v/v) and Tris-HCl pH 7.5 buffer 50 mM in dH_2_O], and incubated overnight in incubation buffer [Triton X 1% (v/v), Tris-HCl pH 7.5 buffer 50 mM, NaCl 2M, and CaCl_2_ 50 mM in dH_2_O], to facilitate enzymatic breakdown of gelatin by MMP-9. The gel was then stained with 0.5% (w/v) Coomassie blue solution for 1 h at room temperature and destained with destain solution (50% dH_2_O, 40% methanol, and 10% acetic acid) until colourless bands appeared at approximately 84 kDa, the molecular weight of MMP-9.

### Statistical analysis

Statistical analyses were performed using GraphPad Prism 9. Statistical significance was set at *P*<0.05, and data were analysed using a Student's paired two-tailed t-test, and one- or two-way ANOVA with Dunnett’s or Fisher’s least significant difference (LSD) *post-hoc* test as indicated in the figure legends. All experiments include four biological replicates (*n* = 4), and the data are expressed as the mean standard error of the mean (SEM).

## Results

### Ischaemia/reperfusion induces oxidative stress in HTR-8/SVneo trophoblasts

**Figure 2:**
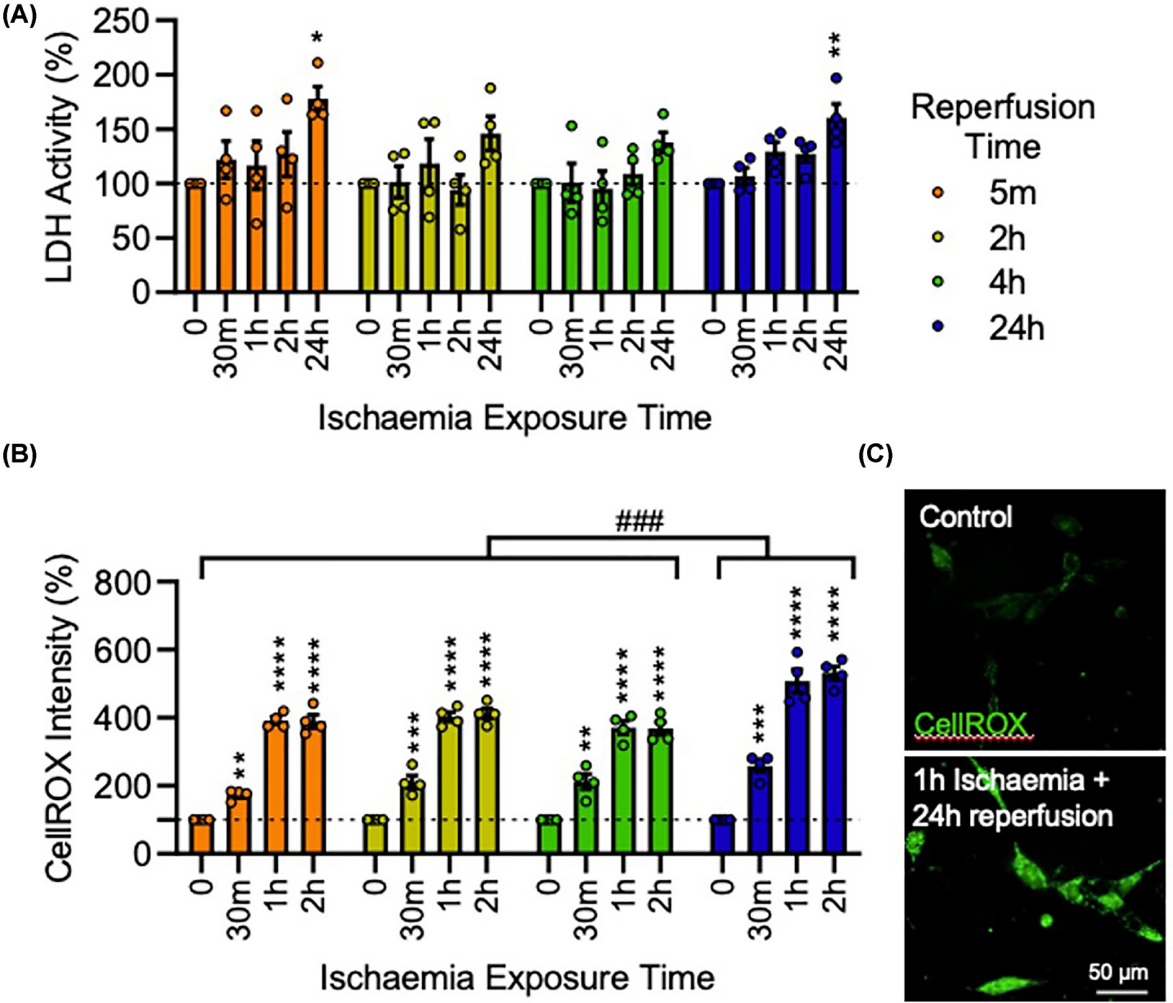
Ischaemia/reperfusion induces oxidative stress in HTR-8/SVneo cells HTR-8/SVneo cells were exposed to simulated ischaemia (SI) and reperfused in complete media for varying durations to characterize and optimize ischaemia/reperfusion exposure times. (**A**) Graph of extracellular LDH activity as a measure of cytotoxicity. (**B**) Graph and (**C**) representative photomicrographs of CellROX™ Green fluorescent intensity as a measure of oxidative stress. Data are mean + SEM from four independent experiments (n=4). Two-way ANOVA with post-hoc Dunnett’s test (* p < 0.05, ** p < 0.01, *** p < 0.001, **** p < 0.0001 vs. Controls; ### p < 0.001 vs. alternative reperfusion times).

We first set out to establish a cellular model of ischemia and reperfusion insult in first trimester cytotrophoblasts which is characteristic of pre-eclampsia. To do this, we used a simulated ischemia buffer which is widely used to model SI/R in studies of cardiac injury [38,40,41] (Figure 1). To characterize and optimize different ischemia and reperfusion insults, HTR-8/SVneo trophoblasts were first exposed to SI and reperfused in complete media for different durations (ischaemia exposure times of either 30 min, 1 h, 2 h, or 24 h; reperfusion times of 5 min, 2 h, 4 h, or 24 h). An increase in cytotoxicity as measured by the release of LDH into the culture medium, was only observed in cells exposed to SI for 24 h (F_4, 60_ = 10.34, *P*<0.0001), with a significant difference specifically between control and 24 h- SI/R-exposed trophoblasts (*P*<0.01), but not for shorter SI exposure times (Figure 2A). This substantial increase in cell death made it impossible to measure intracellular ROS in 24 h- SI-exposed cells. However, even in the absence of increased cell death, SI exposure for 30 min, 1 h, or 2 h all induced a strong oxidative stress response at all reperfusion times (F_3, 48_ = 371.4, *P*<0.0001). Oxidative stress was most substantial after 24 h reperfusion, where 30 min, 1 h, or 2 h SI/R exposure increased ROS levels by 259% (*P*<0.001), 508% (*P*<0.0001), and 531% (*P*<0.0001), respectively (Figure 2B,C). Based on these data, all subsequent experiments used a 1 h ischaemia and 24 h reperfusion protocol. Collectively, these data demonstrate that I/R increases intracellular ROS in trophoblasts almost immediately, and that this is still evident at 24 h.

### Ischaemia/reperfusion induces a transient increase in oxygen consumption rate in HTR-8/SVneo trophoblasts

Having observed an immediate and sustained increase in cellular ROS following I/R, we next sought to determine whether this reflected a change in mitochondrial metabolism by quantifying OCR. OCR was continuously measured in HTR-8/SVneo trophoblasts exposed to 1 h ischaemia and 24 h reperfusion.

**Figure 3:**
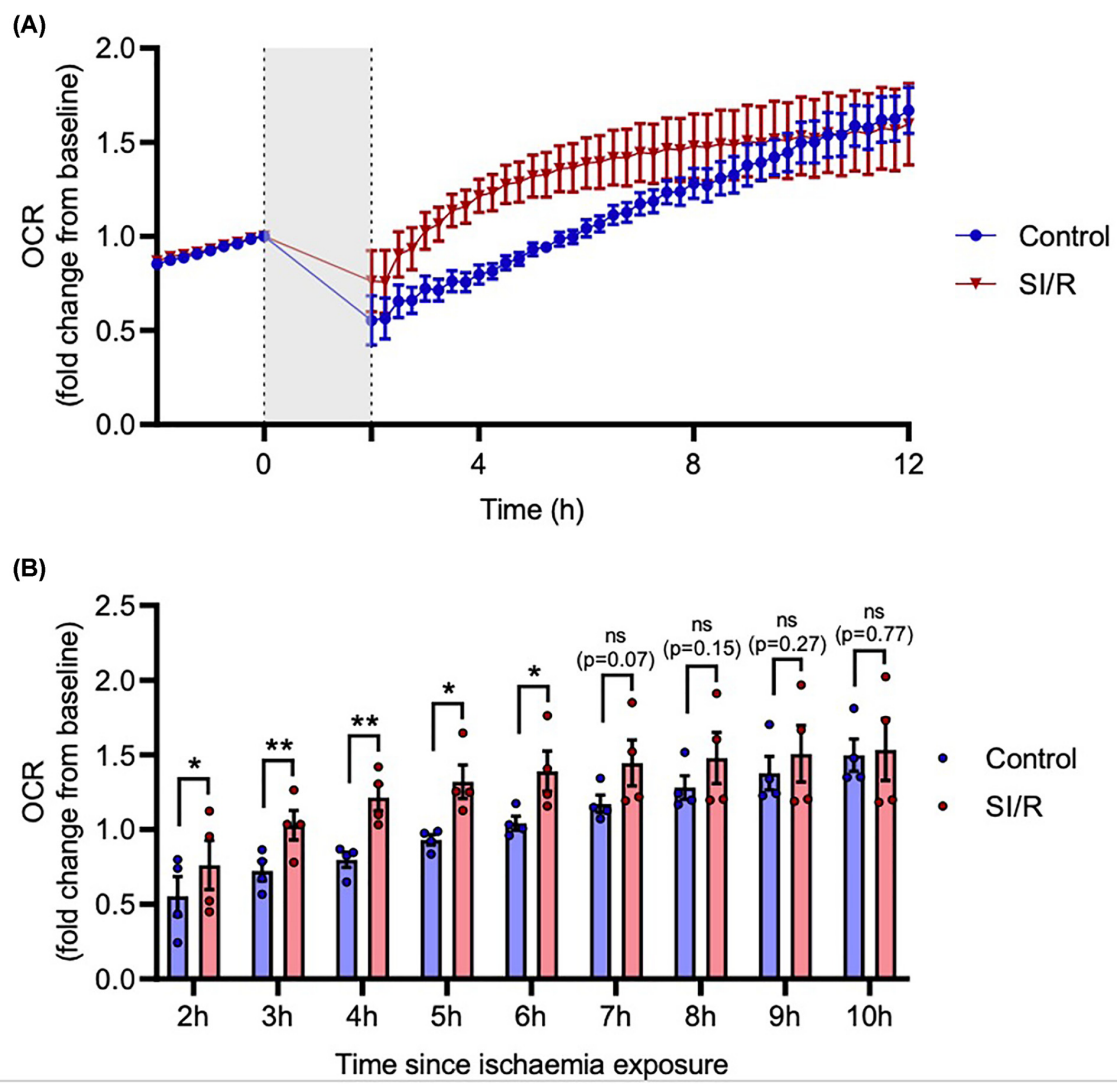
Ischaemia/reperfusion induces a transient increase in oxygen consumption rate in HTR-8/SVneo cells (**A,B**) HTR-8/SVneo cells were exposed to I/R: ischaemia buffer for 1 h and reperfused in complete media for 24 h. (**A**) OCR was measured every 15 min for 24 h using the Resipher system (first 12 h shown). First dashed line represents cessation of baseline recordings, shaded region is 1 h of ischaemia exposure and 1 h of OCR re-equilibration after reperfusion, and second dashed line is resumption of OCR recordings. (**B**) Pair-wise comparisons of OCR at each hour following I/R with data derived from (**A**). Data are mean + SEM from four independent experiments (*n* = 4). Student’s paired t-test (ns = not significant, *P<0.05, **P<0.01 vs. Control).

The normal trend was for OCR to increase linearly over time, but SI/R-exposed cells exhibited a rapid increase in OCR compared to controls, which subsequently returned to a normal respiratory pattern by 12 h (Figure 3A). Hourly increases in OCR were statistically significant from the first available measurement, 2 h after commencement of I/R (t_3_ = 4.345, *P*<0.05), which peaked at 4 h (t_3_ = 6.76, *P*<0.01), but was no longer significant after 7 h (t_3_ = 2.719, *P* = 0.073), and returned to control levels by 10 h (t_3_ = 0.319, *P* = 0.77) (Figure 3B). After this initial spike, OCR continued at a level comparable to controls for 24 h (data not shown).

### The antioxidants N-acetyl cysteine and L-ergothioneine rescue ischaemia-induced oxidative stress in HTR-8/SVneo trophoblasts

**Figure 4:**
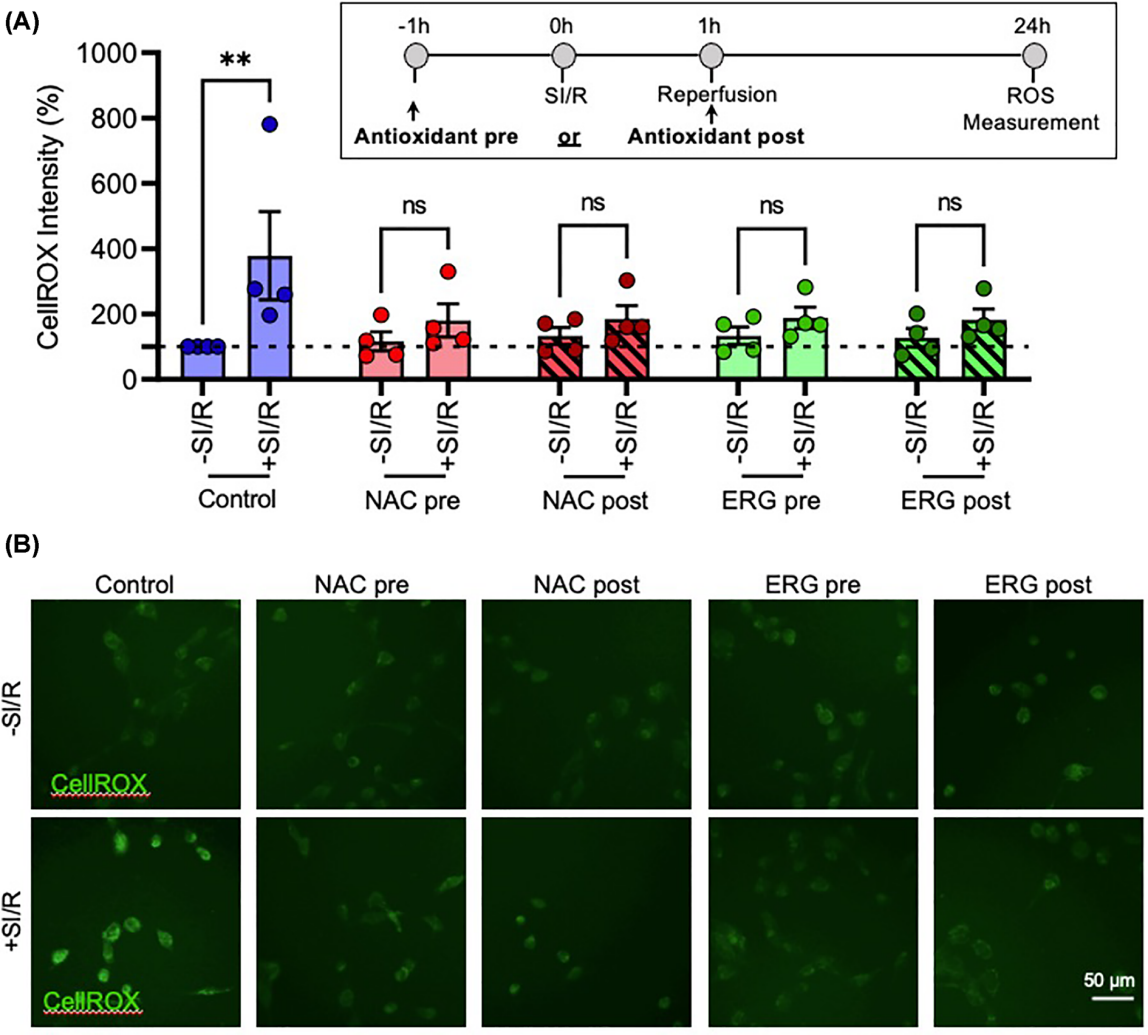
The antioxidants N-acetyl cysteine and L-ergothioneine rescue ischaemia-induced oxidative stress in HTR-8/SVneo cells (**A,B**) HTR-8/SVneo cells were treated with antioxidants 500 μM NAC or 1mM ERG either 1 h before (‘pre-treatment’) or 1 h after (‘post-treatment’) exposure to 1 h ischaemia and 24 h reperfusion. (**A**) Graph and (**B**) representative photomicrographs of CellROX™ Green Reagent fluorescent intensity as a measure of oxidative stress. Data are mean + SEM from four independent experiments (*n* = 4). Student’s paired t-test (ns = not significant, **P<0.01 vs. Control).

To examine whether antioxidant treatment can attenuate this SI/R-induced increase in intracellular ROS, HTR-8/SVneo trophoblasts were exposed to 1 h ischaemia and 24 h reperfusion, and treated with antioxidants 500 μM NAC or 1 mM ERG either 1 h before (‘pre-treatment’) or 1 h after (‘post-treatment’) SI/R exposure was again found to increase intracellular ROS (F_1, 15_ = 12.29, *P*<0.01) but this was not seen in the presence of NAC or ERG (Figure 4A,B). Importantly, the SI/R-induced oxidative stress was attenuated equally by both antioxidants, regardless of whether they were given 1 h pre- or post-ischaemia exposure. Thus, in future experiments only the post-treatment approach was used, due to increased relevance to a treatment scenario.

### Ischaemia/reperfusion impairs HTR-8/SVneo trophoblast migration and proliferation in an oxidative stress-dependent manner

**Figure 5:**
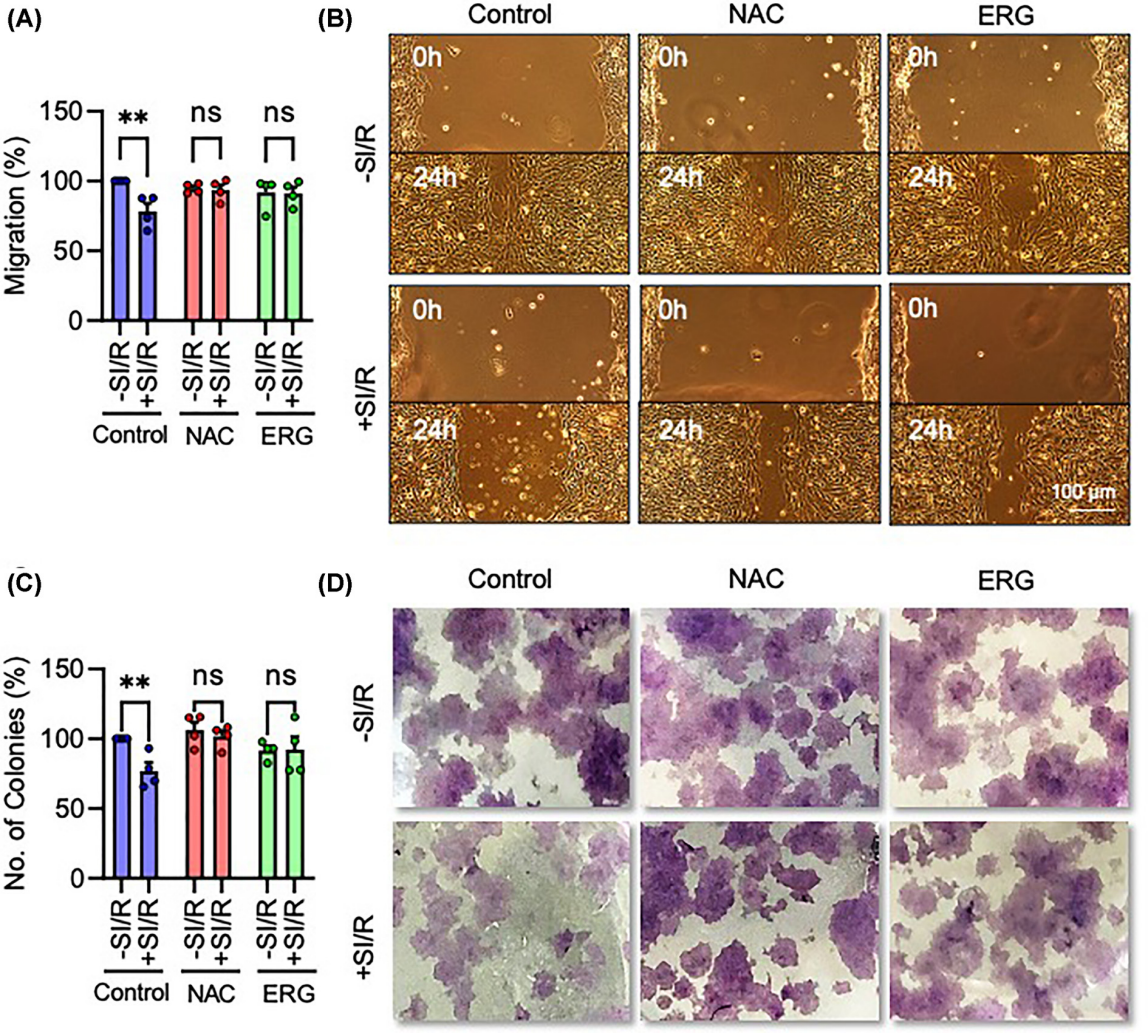
Ischaemia/reperfusion causes oxidative stress-dependent decrease in HTR-8/SVneo cell migration and proliferation (**A-D**) HTR-8/SVneo cells were exposed to simulated ischaemia buffer for 1 h and reperfused in complete media and then treated with antioxidants NAC or ERG. (**A**) Graph and (**B**) representative photomicrographs of cell migration, measured by scratch assay wound closure 24 h after I/R. (**C**) Graph and (**D**) representative photograph of cell proliferation, measured by colony-formation assay 10 days after I/R. Data are mean + SEM from four independent experiments (*n* = 4). Two-way ANOVA and *post-hoc* Fisher’s LSD test (ns = not significant, **P<0.01 vs. Control).

To determine if I/R and the resultant increase in oxidative stress lead to functional deficits in trophoblast cells, we next examined the effects of I/R on migration and proliferation, trophoblast functions which are impaired in PE. HTR-8/SVneo trophoblasts exposed to I/R exhibited decreased migration, measured by scratch assay at 24 h (F_1, 18_ = 5.429, *P*<0.05); which was not seen in cells co-treated with the antioxidants, NAC or ERG (Figure 5A,B). Similarly, I/R reduced HTR-8/SVneo cell proliferation, as measured by a colony formation assay at 10 days (F_1, 18_ = 4.439, *P*<0.05); and the detrimental effects of SI/R on colony formation were rescued by co-treatment with both antioxidants (Figure 5C,D). Thus, I/R impairs trophoblast migration and proliferation, a phenotype that is rescued by antioxidant treatment, suggesting a causal role of oxidative stress in the observed alterations in cellular migration and proliferation.

### Ischaemia/reperfusion impairs HTR-8/SVneo trophoblast invasion, which is independent of oxidative stress and accompanied by reduced MMP-9

**Figure 6:**
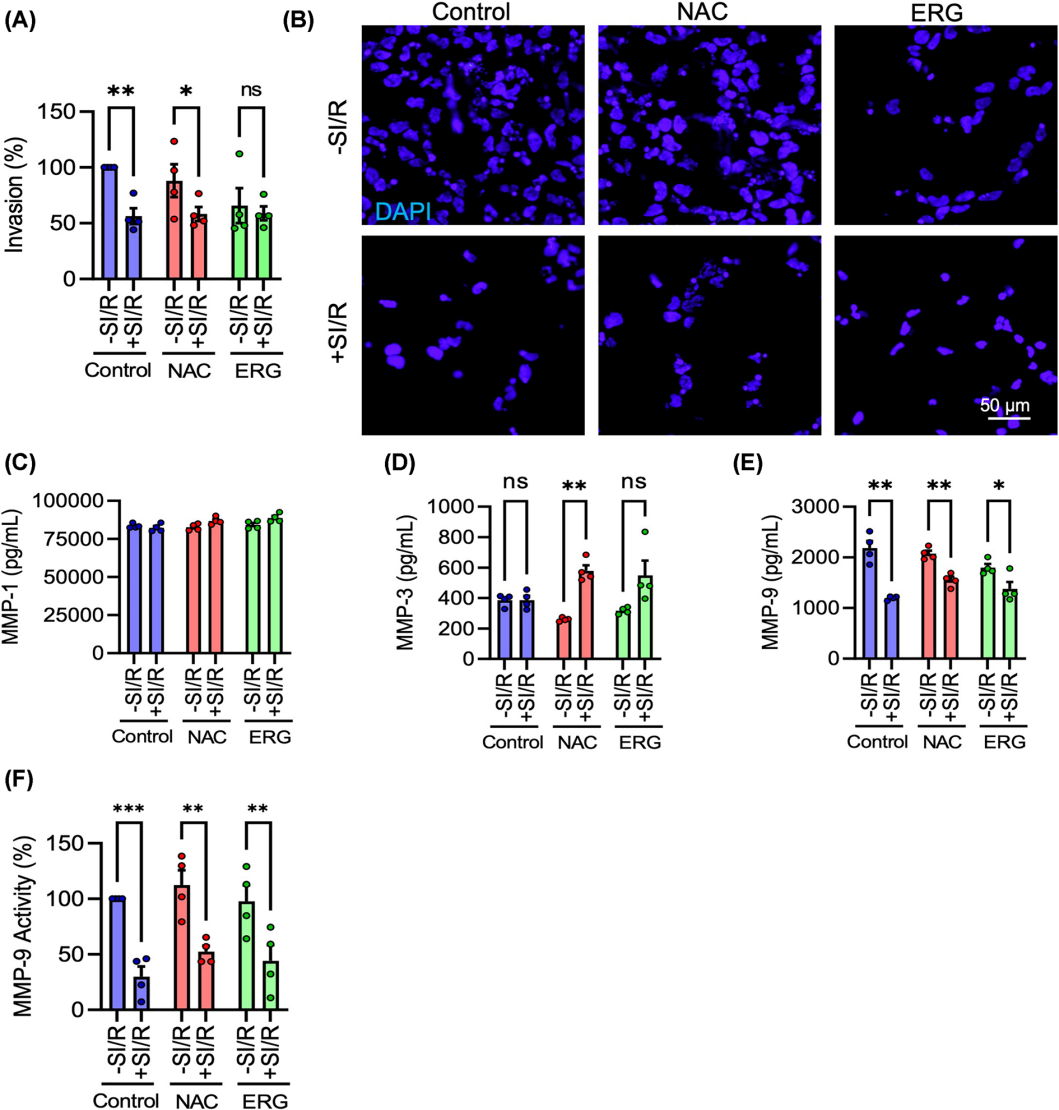
Ischaemia/reperfusion causes oxidative stress-independent decrease in HTR-8/SVneo cell invasion accompanied by reduced MMP-9 activity (**A–F**) HTR-8/SVneo cells were exposed to ischaemia for 1 h and reperfused in complete media and then treated with antioxidants NAC or ERG. (**A**) Graph and (**B**) representative photomicrographs of cell invasion, measured by laminin transwell invasion assay 24 h after I/R. (**C–E**) Extracellular concentrations of MMP-1, MMP-3, and MMP-9, respectively, were measured in conditioned media. (**F**) Extracellular activity of MMP-9 in conditioned media was measured by gelatin zymography. Data are mean + SEM from four independent experiments (*n* = 4). Two-way ANOVA and *post-hoc* Fisher’s LSD test (ns = not significant, *P<0.05, **P<0.01, ***P<0.001 vs. Control).

As well as migration and proliferation, PE is characterised by altered trophoblast invasion [12,13].We thus measured the effects of I/R on HTR-8/SVneo invasion using a laminin transwell invasion assay. I/R impaired trophoblast invasion at 24 h (F_1,6_ = 6.553, *P*<0.05). However, the effect was not rescued by treatment with either antioxidant—the reduced migration was significant under control conditions (*P*<0.01) and in NAC-treated cells (*P*<0.05), while ERG reduced invasion with an effect size equivalent to that of SI/R (Figure 6A,B). This suggests that I/R reduces trophoblast invasion through an alternative, oxidative stress-independent mechanism.

To investigate this further, we measured extracellular levels of MMPs-1, 3, and 9, which are secreted enzymes known to facilitate trophoblast cell invasion by degrading the extracellular matrix [47,48]. MMP-1 concentration was unchanged by I/R (F_1, 6_ = 3.474, *P* = 0.11) (Figure 6C). MMP-3 was similarly unaffected, except unusually in NAC-treated cells, where it was elevated (F_1, 6_ = 14.52, *P*<0.01) (Figure 6D). However, MMP-9 was significantly decreased following I/R insult (F_1, 6_ = 35.99, *P*<0.01), in control (*P*<0.01), NAC-treated (*P*<0.01), and ERG-treated (*P*<0.05) cells (Figure 6E). In support of this decrease in the extracellular concentration of MMP-9, its extracellular *activity* was concomitantly down-regulated, as measured by gelatin zymography (F_1, 18_ = 47.86, *P*<0.0001) in control (*P*<0.0001), NAC-treated (*P*<0.01), and ERG-treated (*P*<0.01) cells (Figure 6F). Thus, neither the extracellular concentration nor activity of MMP-9 was rescued by antioxidant treatment. These data provide strong evidence that I/R causes an oxidative stress-independent decrease in extracellular MMP-9, a key regulatory enzyme of trophoblast invasion.

### MMP-9 regulates HTR-8/SVneo trophoblast invasion, but not migration or proliferation

**Figure 7:**
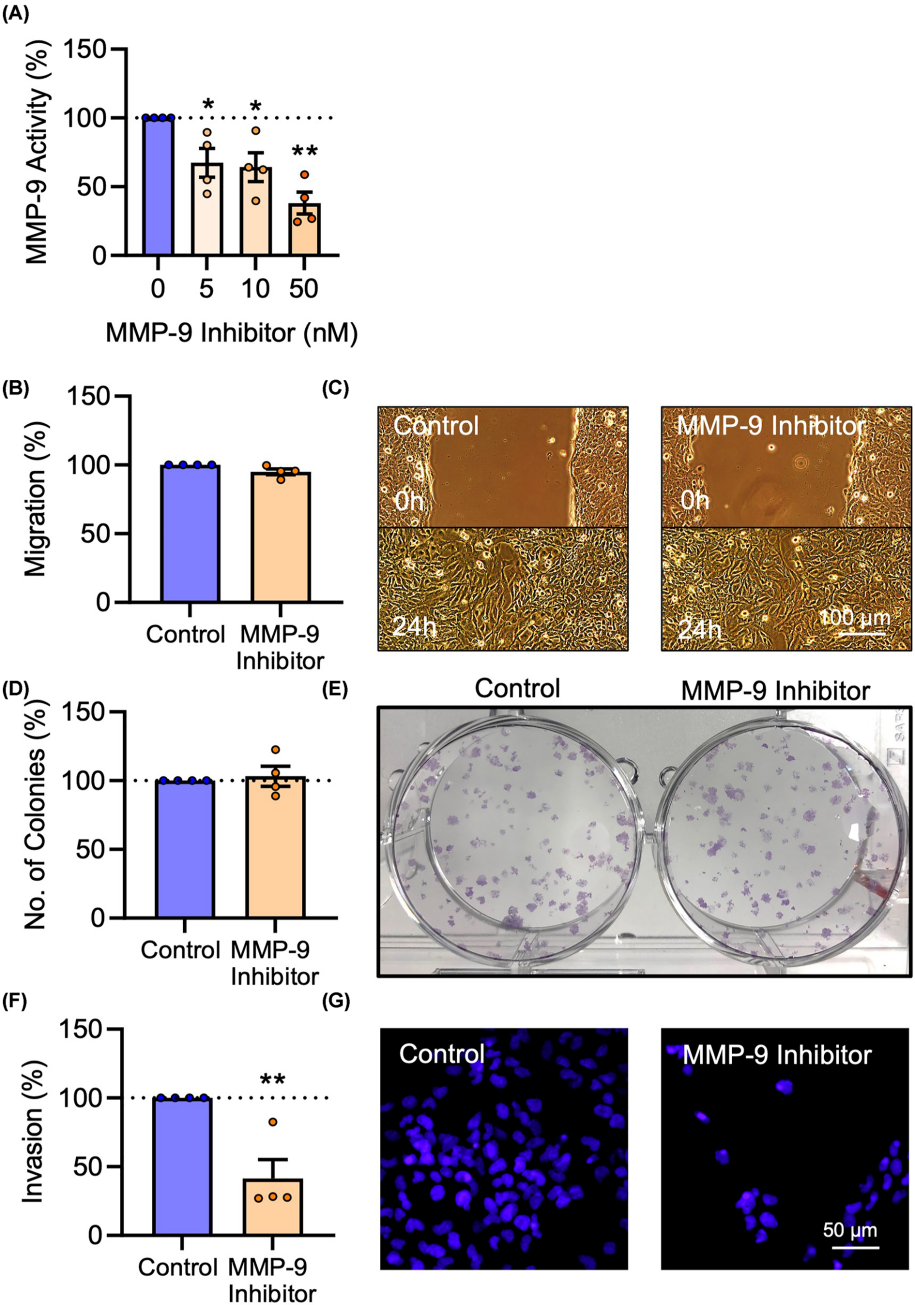
MMP-9 regulates HTR-8/SVneo cell invasion, but not migration or proliferation (**A**) The MMP-9 inhibitor was validated for its ability to inhibit extracellular activity of MMP-9 at 5, 10, and 50 nM by gelatin zymography. (**B–G**) HTR-8/SVneo cells were treated with 10 nM MMP-9 inhibitor or equal volume DMSO as a vehicle control. (**B**) Graph and (**C**) representative photomicrographs of cell migration 24 h after MMP-9 inhibitor treatment. (**D**) Graph and (**E**) representative photograph of cell proliferation 10 days after MMP-9 inhibitor treatment. (**F**) Graph and (**G**) representative photomicrographs of cell invasion 24 h after MMP-9 inhibitor treatment. Data are mean + SEM from four independent experiments (*n* = 4). Student’s paired t-test (*P<0.05, **P<0.01 vs. Control).

Finally, to investigate whether the SI/R-induced reduction of MMP-9 levels could account for its impairment of trophoblast invasion, HTR-8/SVneo trophoblasts were treated with a pharmacological inhibitor of MMP-9 (CAS 1177749-58-4). To first validate its efficacy against extracellular MMP-9 activity, conditioned media samples were treated with MMP-9 inhibitor, and the enzymatic activity of MMP-9 was assessed by gelatin zymography. MMP-9 inhibitor successfully reduced extracellular MMP-9 activity (F_3, 12_ = 9.154, *P*<0.01); activity was reduced by 33% (*P*<0.05), 36% (*P*<0.05), or 52% (*P*<0.01), by 5, 10, or 50 nM, respectively (Figure 7A). Cells were subsequently treated with 10 nM MMP-9 inhibitor and assessed for migration, proliferation, and invasion. MMP-9 inhibition did not affect trophoblast cell migration (t_6_ = 2.328, *P* = 0.06) (Figure 7B,C), or proliferation (t_6_ = 0.4396, *P* = 0.676) (Figure 7D,E). However, cell invasion was severely impaired by inhibiting MMP-9 (t_6_ = 4.27, *P*<0.01) (Figure 7F,G). Collectively, these data demonstrate that MMP-9 positively regulates trophoblast invasion, but not migration or proliferation, which suggests two alternative mechanisms of SI/R-induced functional deficits: impaired migration and proliferation, due to oxidative stress, and impaired invasion, as a result of reduced production and activity of MMP-9.

## Discussion

Early-onset PE is known to result from a failure of extravillous cytotrophoblasts to sufficiently invade the uterus and remodel spiral arteries, resulting in placental I/R. Despite extensive research, our current understanding of the effects of I/R and the resultant oxidative stress on trophoblast function, and thus the pathogenesis of PE, remains uncertain. The current study aimed to use a cellular model of I/R in trophoblasts to examine its effects on trophoblast cell migration, proliferation and invasion, and the potential causative mechanisms.

HTR-8/SVneo cells were exposed to a simulated ischaemia buffer and reperfused in normal media for varying durations to optimize timing of a single round of SI/R exposure. Substantial cell death was only observed when cells were exposed for 24 h; shorter ischaemia exposures induced a strong oxidative stress response which was apparent 5 min after reperfusion, and a 5-fold increase in intracellular ROS was seen after 24 h reperfusion. This is consistent with studies showing that I/R induces oxidative stress in other contexts [22], as does hypoxia/reoxygenation in trophoblasts [17,32,33]. All subsequent experiments used a 1 h ischaemia and 24 h reperfusion paradigm. Trophoblasts exposed to this SI/R paradigm had a substantial increase in OCR in the hours following reperfusion. While ischaemia starves the mitochondria, reperfusion is believed to cause acute mitochondrial hyperactivity which leads to excessive ROS production and eventually mitochondrial dysfunction, so this finding is mechanistically consistent with our observed increase in cellular ROS [49]. The elevated OCR peaked 4 h after induction of SI/R, which is further evidence that reperfusion does not ‘rescue’ the ischaemic insult, but instead exacerbates it. Although OCR returned to baseline levels within 10 h, intracellular ROS remained elevated at 24 h, which suggests that trophoblasts remain highly stressed. Overall, the observed production of ROS and concomitant increase in OCR aligns with the pathological hallmark of placental oxidative stress in PE [36,50].

Having established the I/R paradigm, we sought to examine its effects on the functional properties of trophoblasts that are relevant to early-onset PE—migration, proliferation, and invasion. To explore the potential role of ROS, we first validated the antioxidant capacity of NAC and ERG by establishing that they both rescue approximately 80% of SI/R-induced ROS production in HTR-8/SVneo cells. Both antioxidants use alternative mechanisms to sequester intracellular ROS [51,52], and our findings confirm they are effective in the current context. Notably, the reduction in ROS was equivalent whether the antioxidants were given 1 h pre- or post-ischaemia exposure, and therefore only the latter approach was used in further experiments because of increased clinical relevance where antioxidants might be administered after the I/R insult.

SI/R reduced trophoblast migration, proliferation, and invasion. Although no study to date has examined these functions following exposure to SI/R, all have been shown to decrease following hypoxia/reoxygenation in trophoblasts [30–35]. The only previous study that exposed HTR-8/SVneo cells to a simulated ischaemia buffer observed an accumulation/stabilisation of p53 kinase, a known negative regulator of cell proliferation, migration, and invasion [42,53]. In our study, the deficits in migration and proliferation were rescued by antioxidants, suggesting that these functional impairments are ROS-dependent. Thus, while physiological concentrations of ROS regulate many important aspects of placental physiology [50], excessive levels following I/R are deleterious to trophoblast function. Perhaps of even greater significance is the finding that impaired invasion, the most important and well-studied EVT function in the context of PE, was not rescued by either antioxidant. This suggests that I/R impairs trophoblast invasion through a mechanism independent of oxidative stress. One potential explanation for this divergence is that HTR-8/SVneo cells undergo spontaneous EMT to an invasive phenotype that mimics the *in vivo* process, and thus the cultures are composed of two distinct cell populations—proliferative epithelial-like cells, and the invasive mesenchymal-like cells that they differentiate into [54,55], and it may be that both cellular phenotypes are differentially responsive to ROS and antioxidants.

**Figure 8:**
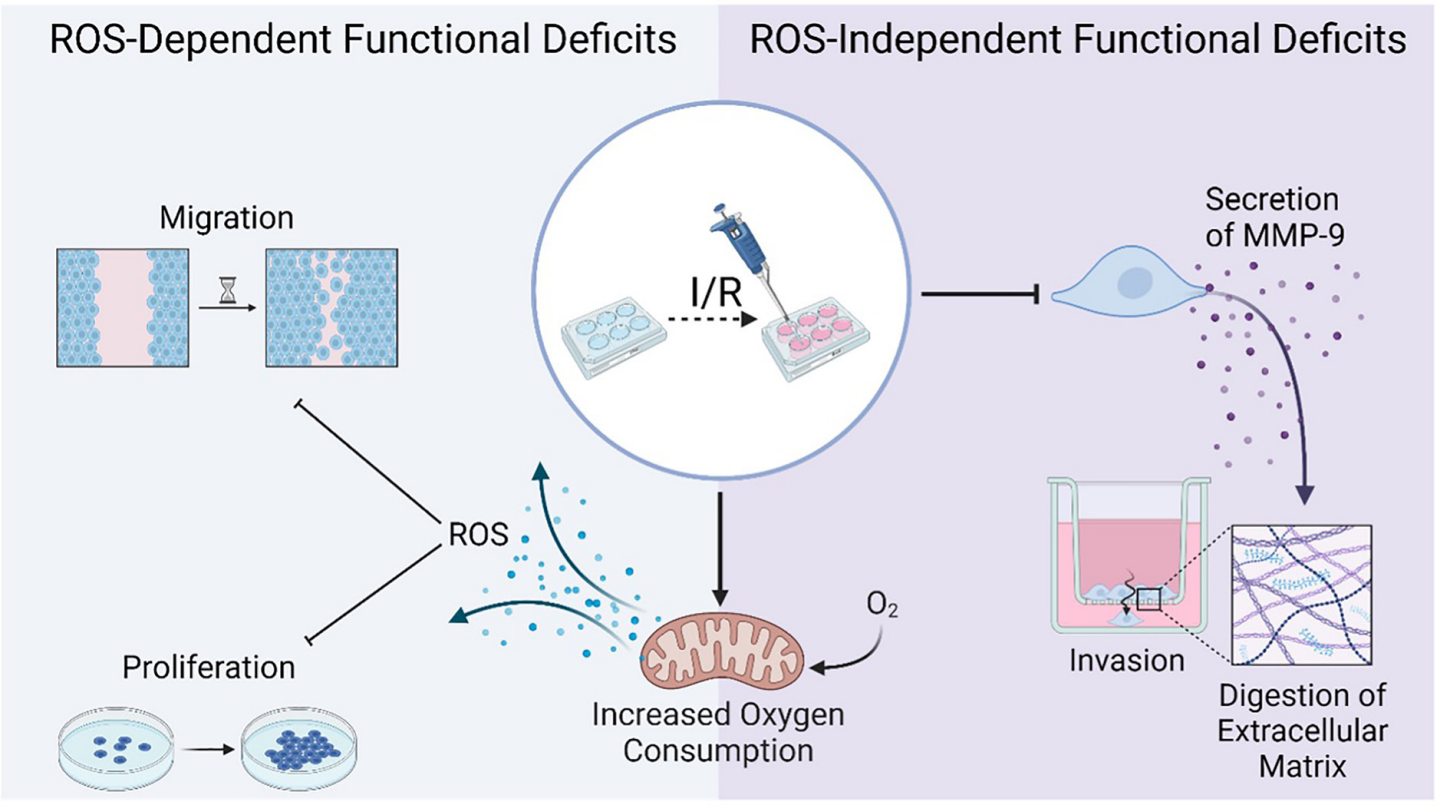
Summary of findings Schematic illustration summarising trophoblast responses to simulated I/R in the current study. Simulated I/R induces two distinct types of functional deficits: ROS-dependent reduction in migration and proliferation, and ROS-independent reduction of MMP-9 and consequently invasion. Figure made using BioRender.com. ROS = reactive oxygen species, I/R = ischaemia/reperfusion.

Having observed that SI/R impaired invasion independent of trophoblast induced-ROS, we next explored an alternative mechanism that may be responsible for defective invasion by focusing on MMPs, which are extracellularly secreted enzymes known to facilitate trophoblast invasion by digesting the uterine extracellular matrix [47]. We observed that SI/R reduces extracellular concentration and activity of MMP-9, a key regulatory enzyme in cell invasion previously reported to be down-regulated in both human PE [34] and hypoxia/reoxygenation-exposed trophoblasts [31,35]. As with the loss of invasion, this reduction in MMP-9 concentration and activity was not attenuated by antioxidants, providing further evidence that the impaired invasion phenotype is not oxidative stress-dependent. Lastly, to determine whether loss of MMP-9 activity is sufficient to mimic the I/R phenotype, we pharmacologically inhibited its extracellular activity and observed a substantial reduction in cell invasion, without any observable deficit in cell migration or proliferation. This is in line with a previous report that depletion of MMP-9 impairs invasion in trophoblast-like BeWo and JEG3 cells [56]. Furthermore, it supports our hypothesis that I/R impairs trophoblast function through two alternative mechanisms: a ROS-dependent decrease in migration and proliferation, and a deficit in invasion mediated by reduced MMP-9 production (Figure 8).

A significant strength of this work is the proof of principle of using this model of SI/R in placental biology and its physiological relevance to the pathological events seen in PE. It is a novel inexpensive, flexible, and reproducible model, optimised here for different ischaemia and reperfusion times. In addition, the use of two antioxidants with different mechanisms of action but the same net effect has allowed us to elucidate the contribution of ROS to I/R-induced functional deficits. Nevertheless, there are some limitations to this work, namely the use of atmospheric (21%) as opposed to physiological (2–5%) O_2_, and the absence of trophoblast–decidual–myometrial interactions that cannot be recapitulated *in vitro*. Additionally, characterisation of the effect of SI/R in other non-EVT trophoblast cell lines such as BeWo, JEG-3 is required to assess its impact on abnormal trophoblast differentiation, a key contributor in PE pathology [57].We have shown here the effect of a single round of SI/R, and it will be of great interest in future to characterise the effect of multiple SI/R challenges, which would be more physiologically reflective of PE. An important question that remains is the elucidation of the cell signalling pathways that mediate the divergent ROS-dependent and ROS-independent responses to I/R. These may include regulation by ROS of redox-sensitive transcription factors such as NF-κB [58], mitochondrial dysfunction, and downstream intercellular signalling by mitochondrial damage-associated molecular patterns [59,60], pathways that converge on transcription factors such as AP-1 and sp-1 that modulate MMP-9 expression [61] and the effects on the homeostatic balance of tissue inhibitors of metalloproteinases (TIMPs) as potential mediators of MMP-9 activity [62]. It is most likely, however, that these signalling cascades and others may be involved.

Overall, we have shown here that while I/R impairs trophoblast migration and proliferation via a ROS-dependent mechanism, the deficiency in invasion was independent of oxidative stress, which may provide one explanation as to why large scale antioxidant trials failed to prevent the incidence of PE. The model we describe here will equally be useful to future researchers studying the molecular pathways involved in the trophoblast response to I/R, and to disambiguate our current understanding of the role of oxidative stress in the pathogenesis of PE.

## Data Availability

All data generated during this study are included in this article and all datasets from which conclusions are based are available on reasonable request from the corresponding authors.

## Abbreviations

BCA: Bicinchoninic acid
EMT: Epithelial to mesenchymal transition
ERG: Ergothioneine
EVT: Extravillous cytotrophoblast
I/R: Ischaemia/reperfusion
LDH: Lactate dehydrogenase
LSD: Least significant difference
MMP: Matrix metalloproteinase
NAC: N-acetyl cysteine
OCR: Oxygen consumption rate
PAGE: Polyacrylamide gel electrophoresis
PE: Pre-eclampsia
PFA: Paraformaldehyde
ROS: Reactive oxygen species
SEM: Standard error of the mean
SI/R: Simulated ischaemia/reperfusion
TIMP: Tissue inhibitors of metalloproteinase
